# Galectins at the maternal-fetal interface: shield or gateway to infections?

**DOI:** 10.3389/fcimb.2026.1960770

**Published:** 2026-10-20

**Authors:** Gabriela Domingues Mattos, Nátalli Zanete Pereira, Maria Notomi Sato

**Affiliations:** 1 Laboratório de Investigação em Dermatologia e Imunodeficiências LIM56, Instituto de Medicina Tropical, Faculdade de Medicina FMUSP, Universidade de São Paulo, São Paulo, SP, Brazil; 2 Departamento de Imunologia, Instituto de Ciências Biomédicas, Universidade de São Paulo USP, São Paulo, SP, Brazil; 3 Departamento de Dermatologia, Faculdade de Medicina FMUSP, Universidade de São Paulo, São Paulo, SP, Brazil

**Keywords:** CMV, gals, HIV, immunity, placenta, ZIKV

## Abstract

Pregnancy relies on a tightly regulated immune balance at the maternal-fetal interface, where tolerance toward the semi-allogeneic fetus must coexist with effective defense mechanisms against invading pathogens. In this context, galectins (gals), a family of β-galactoside-binding lectins, play essential roles in placentation, angiogenesis, tissue remodeling, and immune regulation. Beyond their physiological functions, accumulating evidence indicates that these proteins also participate in host-virus interactions, acting either to restrict infection or to facilitate viral attachment, entry, replication, and persistence. In this review, we discuss the structural characteristics, unconventional secretion pathways, and major immunological functions of gal, with particular emphasis on gal-1, gal-3, gal-9 and gal-13, at the maternal-fetal interface. We also summarize the current evidence regarding their involvement in congenital infections caused by cytomegalovirus (CMV), Zika virus (ZIKV), and human immunodeficiency virus (HIV). Available studies indicate that gals modulate key processes, including viral entry, immune cell activation, inflammation, viral persistence, and maternal-fetal transmission, although their specific mechanisms of action within the placenta remain poorly understood. Collectively, the available evidence suggests that gals exert dual and context-dependent functions, acting as critical regulators of the delicate balance between immune tolerance and antiviral defense. A better understanding of these mechanisms may contribute to the identification of novel biomarkers and improve our understanding of the pathogenesis of congenital infections, ultimately leading to better pregnancy outcomes.

## 1 Immune balance during pregnancy

Pregnancy is a complex biological process that involves major immunological challenges due to the semi-allogeneic nature of the fetus and the need to protect both mother and fetus against invading pathogens (Alberca et al., 2020). In this context, the placenta, plays a central role in ensuring pregnancy success (Cindrova-Davies and Sferruzzi-Perri, 2022). The concept of maternal-fetal tolerance was first proposed by Peter Medawar, a Brazilian-born immunologist (Ribatti, 2015). Medawar suggested that the maternal immune system develops a state of immunological tolerance that allows fetal development and maintenance while preventing immune rejection of this genetically distinct tissue (Rendell et al., 2020).

Structurally, the placenta consists of two major components: the fetal compartment, represented by the chorionic villi, and the maternal compartment, known as the decidua (O'Brien and Wang, 2023). The decidua acting in a coordinated manner, these immune populations are essential for maternal-fetal tolerance, spiral artery remodeling, and successful pregnancy maintenance (Burton et al., 2016; Yang et al., 2020).

During the first trimester, the maternal-fetal interface exhibits a homogeneous immune landscape in terms of both cellular composition and function (La Cour Freiesleben et al., 2021). Decidual NK cells constitute the predominant immune cell population, accounting for approximately 70% of all decidual leukocytes (Sen Santara et al., 2021). They are followed by dendritic cells, decidual macrophages, and regulatory T cells, all of which contribute to the establishment and maintenance of maternal-fetal tolerance (Tong and Abrahams, 2020). In addition, the physical barrier formed by placental cells, the chemical barrier composed of immunoglobulins, chemokines, and cytokines is fundamental to placental immune defense (Nakashima et al., 2021).

In addition to the physical and chemical barriers, maintenance of immune homeostasis at the maternal-fetal interface also depends on soluble glycan-binding molecules. Among these, gals have emerged as key regulators of immune responses at the maternal-fetal interface, contributing to the establishment of tolerance while preserving host defense mechanisms (Oravecz et al., 2022).

## 2 Gals: molecular structure, secretion, binding, and biological functions

Galectins (gals) are a family of endogenous, non-glycosylated proteins first described in 1975 as “galactose-binding lectins” (Johannes et al., 2018). In the placenta, gals play essential roles in placentation, angiogenesis, and the establishment of immune tolerance throughout pregnancy (Balogh et al., 2019; Huang et al., 2021). Among placental gals, gal-1, gal-3, gal-9, gal-13 are of particular interest because of their dynamic roles within the maternal-fetal microenvironment (Blois, 2020). These proteins exhibit remarkable versatility in their cellular localization and can be found as soluble molecules in the cytosol, nucleus, or within extracellular vesicles (García Caballero et al., 2020). Moreover, gals display distinct expression patterns among placental cell populations, including cytotrophoblasts, syncytiotrophoblasts, extravillous trophoblasts, Hofbauer cells, fetal endothelial cells, and decidual immune cells (Chen et al., 2022). Gal-1 is widely expressed in the decidua, endometrium, extravillous trophoblast (EVT), and immune cell populations, including decidual natural killer (dNK) cells and CD4^+^CD25^+^ regulatory T (Treg) cells, as well as in the syncytiotrophoblast (STB). In contrast, gal-3 is predominantly localized in the cytotrophoblast, villous trophoblast, and endometrium. gal-9 is mainly expressed in the cytotrophoblast, decidua, and endometrium, whereas gal-13, also known as placental protein 13 (PP13), is almost exclusively expressed by the syncytiotrophoblast, highlighting its placenta-specific role during pregnancy (Koopman et al., 2003; Von Wolff et al., 2005; Blois et al., 2007; Barrientos et al., 2014; Bojić-Trbojević et al., 2014; Oravecz et al., 2022; Mendoza et al., 2024). Based on their structural organization, gals are classified into three major groups: prototype, tandem-repeat, and chimera-type gals (Bänfer and Jacob, 2020; García Caballero et al., 2020). In contrast, gal-3 is the only chimera-type (Bänfer and Jacob, 2020). The carbohydrate recognition domain (CRD) in gals consists of two antiparallel β-sheets folded into a β-sandwich structure (Wu et al., 2021). The CRD is responsible for differences in ligand specificity structural diversity is particularly evident in gal-3, whose unique architecture provides greater functional flexibility (Thiemann and Baum, 2016; Wu et al., 2021).

To interact with extracellular glycans, gals must first be released from the cell after synthesis (Paz et al., 2010). Unlike most secreted proteins, gals lack a conventional signal peptide, indicating that they are exported through an unconventional secretion pathway that bypasses both the endoplasmic reticulum and the Golgi apparatus (Barondes et al., 1994). Although the classical secretory pathway has been extensively characterized, the molecular mechanisms underlying the unconventional secretion of gals remain incompletely understood in most tissues, highlighting the need for further investigation (Mayo, 2023). Once secreted, gals establish selective and functionally relevant interactions by recognizing glycans predominantly enriched in β-galactosides, which are widely distributed among placental cell types (Mayo, 2023). Although gals preferentially bind β-galactosides, some family members also exhibit affinity for β-mannosides (Balogh et al., 2019). Gals interact with a broad repertoire of receptors and ligands through glycan recognition on the cell surface and within the extracellular matrix (Kaltner et al., 2017). Their major binding partners include integrins, receptor tyrosine kinases, Notch signaling components, neuropilin-1, cell adhesion molecules, several immune receptors, as well as glycosphingolipids such as GM1 (Troncoso et al., 2023).

At the maternal-fetal interface, gals expression is tightly regulated throughout gestation and among distinct placental cell populations (Kirkgöz et al., 2024). Among their best-characterized ligands is MUC1, whose interaction with gal-1 contributes to extravillous trophoblast adhesion, migration, and invasion, thereby supporting placentation (Bojić-Trbojević et al., 2014). Pregnancy-specific glycoprotein 1 (PSG1) also interacts with gal-1 and gal-9, modulating immune responses at the maternal-fetal interface (Mendoza et al., 2024). Additional ligands, including the NRP1/VEGFR-2 receptor complex, CD44, and β1 integrins, further contribute to galectin-mediated signaling in trophoblasts and decidual cells (Cano et al., 2021). The placenta-specific gal-13 e gal-14, remain incompletely characterized with respect to their cellular ligands (Yang et al., 2020). Nevertheless, accumulating evidence associates these gals with key processes involved in vascular adaptation and immune regulation during pregnancy (Huang et al., 2021; Troncoso et al., 2023) (as shown in **Figure 1**).

**Figure 1:**
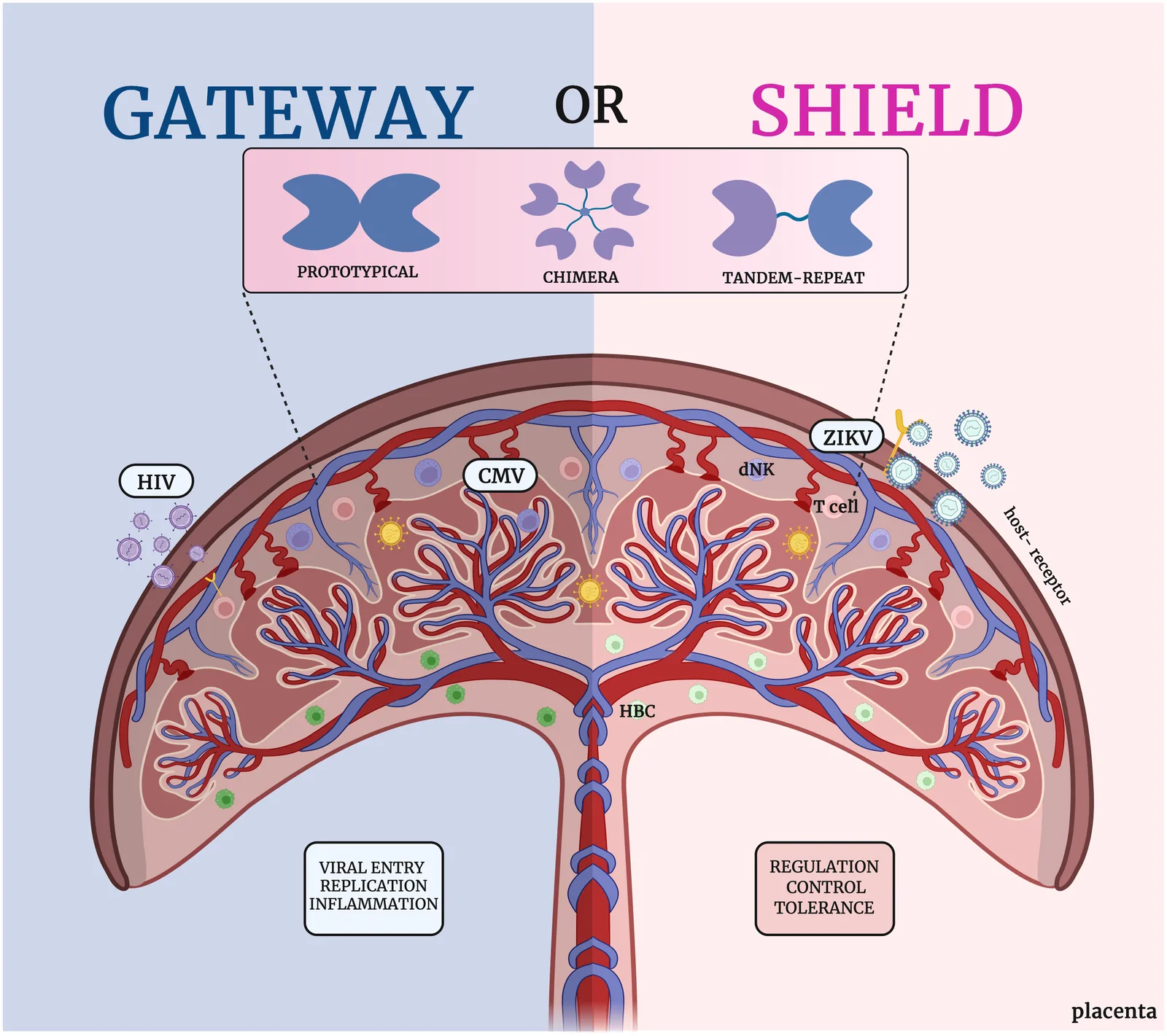
Galectins at the maternal-fetal interface: shield or gateway to infections? Galectins exhibit context-dependent functions at the maternal–fetal interface, acting either as a gateway that facilitates viral attachment, entry, replication, and persistence or as an immunological shield that promotes immune regulation, antiviral defense, and maternal–fetal tolerance. The three structural classes of Galectins (prototype, chimera, and tandem-repeat) are shown at the top. Within the placental microenvironment, Galectins modulate the interactions between trophoblasts, Hofbauer cells (HBCs), decidual natural killer (dNK) cells, T cells, and viral pathogens, including human immunodeficiency virus (HIV), cytomeGalovirus (CMV), and Zika virus (ZIKV). Their biological effects ultimately influence placental homeostasis and the outcome of congenital infections. Created in BioRender. Domingues mattos, G. (2026) https://BioRender.com/p77lir1.

Sialic acid also regulates gal function by masking N-acetyllactosamine (LacNAc) epitopes and thereby modulating gal-glycan interactions (Zhuo and Bellis, 2011). During the first trimester, trophoblasts release highly sialylated antigens that contribute to immune regulation at the maternal-fetal interface (Nakashima et al., 2021; Zhong et al., 2024). Impaired trophoblast sialylation can result in embryonic developmental failure associated with maternal complement activation (Abeln et al., 2019). The type of sialic acid linkage can also influence gal-1 binding to glycans (Zhuo and Bellis, 2011).

Redox conditions, for example, can alter gal structure through the formation of disulfide bonds, whereas glycan binding has been shown to exert a protective effect against oxidative inactivation (Kamili et al., 2016).

## 3 Viruses associated with gals in pregnancy

Interactions with pathogens occur within a physiological context in which the maternal organism undergoes profound immunological and endocrine adaptations throughout pregnancy (Vasta, 2020). Although these adaptations are essential for maintaining tolerance toward the semi-allogeneic fetus, they may also compromise antiviral immune responses, rendering pregnancy a period of increased susceptibility to viral infections (Mor, 2022). Pregnancy-associated hormonal changes contribute to reduced dendritic cell and monocyte activity, impaired macrophage effector function, partial inhibition of natural killer (NK) cell activity, and altered T- and B-cell responses (Teixeira et al., 2020). Accordingly, pregnant women are at increased risk of severe disease and mortality during viral outbreaks, including Ebola virus, influenza, and SARS-CoV-2 infections (Groß et al., 2024). Within this context, several viruses are distinguished not only by their systemic dissemination but also by their ability to infect the maternal-fetal interface. These pathogens may reach the placenta through two principal routes: (i) the ascending route, following disruption of the cervical and reproductive tract barriers, and (ii) the hematogenous route, in which viruses disseminate through the maternal circulation before reaching placental tissues (Fuentes-Zacarías et al., 2021).

### 3.1 Systemic and non-placental evidence of gal-virus interactions

#### 3.1.1 Cytomegalovirus

Human cytomegalovirus (CMV), a member of the Herpesviridae family, is a major congenital pathogen. Vertical transmission may occur following primary maternal infection, reinfection, or reactivation of latent virus during pregnancy (Geris et al., 2022). Unlike many other congenital infections, the risk of fetal involvement during maternal CMV infection is particularly relevant because of the high seroprevalence among women of reproductive age (Britt, 2015). Among these, gals have emerged as key modulators of antiviral immunity through their ability to recognize glycans expressed on both host cells and viral particles (Vasta, 2020). Gal-9 expression is upregulated in human fibroblastsduring HCMV infection *in vitro* and in infected individuals, a phenomenon likely driven by interferons, which are potent inducers of LGALS9 expression during viral infections (McSharry et al., 2014; Machala et al., 2019). Moreover, in an *in vivo* murine model, gal-9-deficient mice infected with murine CMV exhibited exaggerated natural killer cells (NK) activation and enhanced inflammatory responses, suggesting that gal-9 negatively regulates NK-cell activity (Golden-Mason et al., 2013). Similarly, in an *in vivo* murine model of CMV-induced hepatitis, gal-3-deficient mice displayed increased viral replication, exacerbated inflammation, and enhanced TNF-α-mediated cell death, whereas treatment with recombinant gal-3 attenuated liver injury, supporting a protective role for this lectin during CMV infection (Stojanovic et al., 2019).

#### 3.1.2 Zika virus

Among viral infections associated with adverse pregnancy outcomes, Zika virus (ZIKV) represents one of the most devastating pathogens because of its marked neurotropism (Bhagat et al., 2021). ZIKV is a mosquito-borne virus belonging to the Flaviviridae family and, although it is no longer responsible for widespread epidemics, it remains an important public health concern (Giraldo et al., 2023). Despite extensive research efforts following the recent outbreak in the Americas, no licensed vaccine is currently available (De Jong and Grobusch, 2025). Evidence from non-placental systems has demonstrated that gal-1, expressed on the surface of human neural progenitor cells, is an essential component of the viral entry complex by cooperating with the viral receptor AXL (Gao et al., 2022). This interaction was confirmed through antibody-mediated blockade and LGALS1 gene silencing, both of which significantly reduced viral infection. Furthermore, in an *in vivo* murine model, LGALS1^–^/^–^ mice exhibited increased resistance to ZIKV infection, characterized by lower viral loads and reduced virus-induced pathological alterations (Gao et al., 2022).

#### 3.1.3 Human immunodeficiency virus

The etiological agent of acquired immunodeficiency syndrome (AIDS) remains a major global public health concern and poses a significant challenge during pregnancy because of the risk of mother-to-child transmission (Mor, 2022). Unlike CMV and ZIKV, which exhibit tropism for trophoblast cells, HIV reaches the maternal-fetal interface primarily through infected maternal immune cells residing within the decidua (Castillo et al., 2025). HIV infection is strongly influenced by glycan-dependent interactions, making gals important modulators of both antiviral immune responses and viral dynamics (St-Pierre et al., 2011). Within this context, gal-1 exerts complex effects during HIV-1 infection. Experimental studies have demonstrated that gal-1 enhances viral attachment by bridging the viral envelope glycoprotein gp120 to the CD4 receptor, thereby increasing viral infectivity (Mercier et al., 2008). Gal-1 also contributes to the reactivation of latent viral reservoirs through activation of the NF-κB signaling pathway (Rubione et al., 2022). Similarly, gal-3 promotes cell-to-cell viral dissemination and viral replication, since inhibition reduces NF-κB activation and viral particle production. Elevated circulating levels of gal-3 have also been reported in people living with HIV, reflecting the persistent inflammatory state associated with chronic infection (Wang et al., 2022; Vares-Lum et al., 2023). Gal-9 also contributes to HIV-1 immunopathogenesis by simultaneously regulating viral transcription and host immune responses (Colomb et al., 2019). Increased gal-9 expression has been associated with enhanced HIV transcription in CD4^+^ T cells during antiretroviral therapy and with the reactivation of latent viral reservoirs (Colomb et al., 2019).

### 3.2 Evidence at the maternal-fetal interface

#### 3.2.1 Cytomegalovirus

CMV exhibits tropism toward trophoblast cells and replicates efficiently in cytotrophoblasts (Geris et al., 2022). CMV infection can induce placental edema and fibrosis, impairing oxygen and nutrient transport to the fetus (Tabata et al., 2015) and ultimately contributing to fetal growth restriction (Tabata et al., 2015). Despite these findings, the role of gals at the maternal-fetal interface during CMV infection remains poorly understood. In this context, proteomic analysis of amniotic fluid from fetuses with congenital CMV infection identified elevated levels of gal-3-binding protein (gal-3BP), an immunomodulatory glycoprotein that directly interacts with gal-3 (Vorontsov et al., 2022). Increased gal-3BP concentrations were associated with severe congenital CMV, suggesting that gal-3-related pathways may contribute to disease pathogenesis and severity (Vorontsov et al., 2022).

#### 3.2.2 Zika virus

Following maternal infection, vertical transmission of ZIKV may occur during pregnancy or the perinatal period (Barrozo et al., 2024). However, infection during the first trimester is associated with severe fetal injury (Cardim et al., 2025). Congenital Zika Syndrome (CZS) is characterized by a spectrum of abnormalities observed in infants exposed to the virus *in utero*, including defects affecting the central nervous, musculoskeletal, and visual systems (Crisanto-López et al., 2023). Nevertheless, the contribution of placental infection to the development of CZS remains incompletely understood, as ZIKV is also capable of directly infecting the fetal central nervous system (Crisanto-López et al., 2023).

During pregnancy, ZIKV is thought to reach the placenta primarily through infection of the decidua, since maternal decidual cells remain permissive to viral infection throughout gestation (Tabata et al., 2015; Weisblum et al., 2017). ZIKV infects cytotrophoblasts and extravillous trophoblasts during the first trimester, whereas syncytiotrophoblasts remain largely resistant throughout pregnancy (Tabata et al., 2015). Following infection, anti-inflammatory mediators and antiviral pathways, particularly the interferon signaling pathway, are activated (Grant et al., 2016). Within this context, although still limited, emerging evidence suggests that gals participate in modulating the immune response to ZIKV infection. More recently, a Brazilian study analyzing 45 placentas from women infected with ZIKV demonstrated that placentas from newborns with congenital Zika syndrome exhibited marked histopathological alterations, including villous edema, coagulative necrosis, and trophoblast detachment (Leme, 2021). In the same study, gal-3 was markedly overexpressed in cytotrophoblasts, where it colocalized with the viral antigen NS2B, accompanied by Hofbauer cell hyperplasia and increased proliferative activity of both Hofbauer cells and trophoblasts (Leme, 2021). Moreover, transcriptomic analysis performed at the University of São Paulo using trophoblasts derived from discordant twins for congenital Zika syndrome revealed significant downregulation of LGALS3 following ZIKV infection (Amaral et al., 2020). Despite these findings, the molecular mechanisms linking gals to placental ZIKV infection remain poorly understood, highlighting the need for further mechanistic studies at the maternal-fetal interface.

#### 3.2.3 Human immunodeficiency virus

Increased concentrations of gal-3-binding protein (gal-3BP) in breast milk have been associated with higher maternal viral load and an increased risk of postnatal HIV transmission, highlighting its potential as a biomarker of mother-to-child transmission (Chan et al., 2013). Moreover, elevated serum gal-9 levels have been correlated with poorer cognitive performance in adolescents infected through vertical transmission (Elahi, 2024).

## 4 Discussion

Despite significant advances in understanding the roles of gal-1, gal-3, and gal-9 during viral infections, important gaps remain regarding their functions at the maternal-fetal interface. In particular, gal-13 remains one of the least investigated placental gals. Although associated with maternal-fetal immune tolerance, placental remodeling, and pregnancy maintenance, limited evidence directly links gal-13 to viral infections or vertical transmission (Yang et al., 2020). Thus, its role during congenital viral infections remains largely undefined. The available evidence also indicates that gal effects on viral infection are highly context-dependent, as illustrated by the contrasting activities summarized in **Table 1**.

**Table 1:** Dual role of gals in viral infections at the maternal-fetal interface: shield or gateway?

Virus	Gals	Effect	Mechanism	Outcome	References
CMV	Gal- 9	Antiviral	Upregulated by IFNs; regulates NK cell activation	Controls excessive inflammation; prevents overactivation	(Golden-Mason et al., 2013; Machala et al., 2019)
CMV	Gal-3	Protective	Reduces TNF-α-mediated cell death and inflammation in mouse model	Attenuates tissue damage	(Stojanovic et al., 2019)
CMV	Gal-3BP	Biomarker	Interacts with Gal-3; elevated in severe infection	Associated with severe congenital CMV	(Vorontsov et al., 2022)
ZIKV	Gal-1	Proviral	Facilitates viral entry via interaction with AXL receptor	Increases viral entry and replication	(Gao et al., 2022)
ZIKA	Gal-3	Biomarker	Hyperplasia of Hofbauer cells and increased proliferative activity of both Hofbauer cells and trophoblasts	Associated with SCZ	(Leme, 2021)
HIV	Gal-1	Proviral	Bridges gp120 and CD4, enhancing viral attachment	Increased viral entry and infectivity	(Mercier et al., 2008; St-Pierre et al., 2011)
HIV	Gal-1	Proviral	Activates NF-κB reactivation of latent virus	Promotes viral reactivation	(Rubione et al., 2022)
HIV	Gal-3BP	Biomarker	Elevated in breast milk; correlates with viral load	Associated with postnatal transmission	(Chan et al., 2013)
HIV	Gal-3	Proviral	Enhances cell-to-cell viral transmission via lipid raft modulation	Increased viral dissemination	(Wang et al., 2022)
HIV	Gal-3	Antiviral	Induces death of infected macrophages	Limits infected cell survival	(Xue et al., 2017)
HIV	Gal-9	Dual role	Activates ERK/CREB pathways	Promotes viral transcription inflammation	(Colomb et al., 2019)
HIV	Gal-9	Proviral	Reactivates latent reservoirs	Increases viral transcription	(Adeniji et al., 2022)
HIV	Gal-9	Biomarker	Correlates with cognitive impairment and inflammation	Associated with disease progression	(Elahi, 2024)

The CMV findings illustrate this context dependence. Systemic studies showed that gal-9 regulates NK-cell activation during murine CMV infection, suggesting a role in limiting inflammatory responses (Golden-Mason et al., 2013). However, whether this mechanism operates at the maternal-fetal interface remains unknown. Decidual NK (dNK) cells are the predominant leukocyte population in early pregnancy and differ functionally from peripheral NK cells (Moffett-King, 2002). Because gal-9 interacts with TIM-3, this axis represents a potential mechanism through which gal-9 could regulate dNK-cell responses during placental infection. Future studies should determine whether gal-9/TIM-3 interactions influence dNK-cell activation, cytokine production, and antiviral responses, and whether this regulation primarily limits tissue inflammation or compromises antiviral defense. ZIKV provides another example of this context dependence. In placentas from women infected with ZIKV, increased gal-3 expression in cytotrophoblasts, colocalization with viral NS2B, and Hofbauer cell hyperplasia were associated with congenital Zika syndrome (Leme, 2021). Conversely, trophoblasts derived from discordant twins showed downregulation of LGALS3 following ZIKV infection (Amaral et al., 2020). These apparently divergent findings may reflect differences between tissue-level responses and cell-intrinsic regulation. Importantly, the association between gal-3 expression and Hofbauer cell hyperplasia raises the possibility that gal-3 contributes to the placental response to infection, although functional studies are required to determine whether this represents a protective response or a consequence of infection-associated tissue remodeling.

Taken together, current evidence does not support a universal classification of gals as either antiviral or proviral molecules. Rather, their effects appear to depend on the specific gal, virus, target cell, and local glycan and immune environment. At the maternal-fetal interface, this context dependence may be particularly important because mechanisms that normally promote immune tolerance and tissue homeostasis could also influence viral persistence or dissemination. The available evidence across CMV, ZIKV, and HIV therefore highlights a common gap: although gals have been associated with viral infection, direct functional evidence at the maternal-fetal interface remains limited. Future studies combining single-cell and spatial approaches with placental organoids, decidual immune-cell systems, and functional infection models will be essential to define the cell-specific functions of gals during congenital infections. In particular, functional studies should distinguish whether altered gal expression is a consequence of infection or directly affects viral entry, replication, immune responses, trophoblast function, and Hofbauer cell responses. Such studies may also clarify the potential of gals as biomarkers of congenital infection and disease severity. Therefore, defining whether gals, particularly poorly characterized placental gals such as gal-13, contribute to antiviral defense or are exploited by viruses remains an important challenge. At the maternal-fetal interface, do gals function primarily as an immunological shield or can they also become a gateway for viral invasion?
